# Assessing the Use, Impact, and Perspectives of Generative Artificial Intelligence Among Health Consumers: A Cross-Sectional Survey in Saudi Arabia

**DOI:** 10.3390/healthcare14213558

**Published:** 2026-10-22

**Authors:** Mohammed A. Alateeq, Mohammad A. Alruhaimi, Abdulmalik K. Alismail, Mohammed S. Alanazi, Amer A. Alameer, Rayan Alharbi, Nawaf O. Almutairi, Bader M. Alshoumr

**Affiliations:** 1 Ambulatory Care Services, Ministry of National Guard—Health Affairs, Riyadh 11426, Saudi Arabia; 2 King Abdullah International Medical Research Center, Riyadh 11481, Saudi Arabia; 3 College of Medicine, King Saud Bin Abdulaziz University for Health Sciences, Riyadh 12372, Saudi Arabia; alruhaimi.mohammad@gmail.com (M.A.A.); aka.alismail@gmail.com (A.K.A.); alanazimbs@gmail.com (M.S.A.); amer.alameer1@icloud.com (A.A.A.); alharbir0111@gmail.com (R.A.); 4 Department of Health Informatics, College of Public Health and Health Informatics, University of Ha’il, Ha’il 55476, Saudi Arabia; n.almutairi@uoh.edu.sa (N.O.A.); b.alshoumr@uoh.edu.sa (B.M.A.); 5 Medical and Diagnostic Research Center, University of Ha’il, Ha’il 55476, Saudi Arabia

**Keywords:** health consumer, online health information, artificial intelligence, Saudi Arabia, consumer health informatics

## Abstract

Background/Objectives: Saudi Health consumers increasingly rely on generative Artificial Intelligence (AI) to access health information from a growing number of health-related resources. Little is known about the frequency and patterns of generative AI tool use for seeking health information among consumers, its reported impact, and health consumers’ perceptions of these tools. This study aims to investigate the use of and perspectives on generative AI tools among health consumers in Saudi Arabia (SA). It also aims to assess the impact of their use on health-seeking behaviours. Methods: An exploratory, cross-sectional, self-administered online survey was distributed to adults (≥18 years) residing in SA during two data collection rounds in 2026 (March–April and July–August). All adults who used generative AI tools to seek online health information in the past year were included. Bivariate and multivariate analyses were conducted to examine relationships among variables. Results: A total of 403 eligible health consumers (58% male) participated. Among respondents who had used generative AI for health purposes, approximately 49% reported using it more than five times per month. Younger age, higher income, and more frequent pre-use were associated with higher odds of frequent seeking health information behaviour. Headache, abdominal pain, and musculoskeletal pain were the most common claims. ChatGPT was the main platform (84%) used for seeking information. Age was significantly negatively associated with frequency, whereas income and pre-visit use were positively associated. The primary reasons for seeking health information via generative AI were quick information retrieval (71%), time saving (34%), and curiosity (30.5%). Conclusions: The high frequency of use suggests that generative AI can serve as a source of health information and may lead users to withhold health information from healthcare providers. It may influence subsequent health-seeking behaviours, such as requesting urgent care or postponing physician visits. These self-reported findings do not establish diagnostic accuracy, clinical effectiveness, or patient safety, and the clinical risk and appropriateness of self-management actions were not assessed. Many respondents reported concerns about inaccurate information, missed serious illness, and increased health anxiety.

## 1. Background

The physician–patient encounter has long been the primary entry point for medical decision-making. Traditionally, health consumers experiencing illness sought guidance from trusted clinicians, reserving self-initiated action for minor or transient symptoms. The widespread accessibility of the internet has led many health consumers to seek health information online before consulting a physician, a behavior commonly termed “consulting Dr. Google” [1]. This reflects a broader global shift toward seeking health information online; over 80% of internet users have engaged in online health searches at least once [2]. In response, generative Artificial Intelligence (AI) tools have emerged to support conversational clinical triage [3].

Using generative AI tools to obtain health recommendations provides instant, affordable access to health-related content for health consumers, improving healthcare decision-making and better preparing them for upcoming medical encounters. Consumers’ characteristics, health status, employment status, and geographic location influence the prevalence and extent of online health-seeking behaviour [2,4]. However, the use of generative AI can lead to misinformation or false health-related conclusions. Drawbacks may include affecting consumers’ decision-making, preventing timely medical care, causing serious medical harm or adverse events, or leading to unnecessary healthcare services or visits [5,6].

Seeking online health information from generative AI tools may affect the quality of the physician–patient relationship and pose distinct challenges during the clinical encounter [7,8]. Several studies show that this behaviour may influence patients’ acceptance of clinical recommendations and adherence to treatment [9,10,11]. Identifying credible sources and accessing alarming or inaccurate content may exacerbate health-related anxiety—a phenomenon referred to as cyberchondria [12,13]. As generative AI increasingly addresses medical queries [14], its diagnostic performance has been noteworthy. One study showed that one generative AI tool (i.e., ChatGPT-3) achieved 88% diagnostic accuracy, substantially exceeding that of laypersons (54%) but remaining below the 96% accuracy attained by physicians [15].

Although generative AI tools can provide immediate, technically reliable responses, they remain limited in replicating the relational trust that underpins the physician–patient relationship. This lack of trust is deeply rooted in the interpersonal and human dimensions of care. Therefore, AI’s technical capabilities alone cannot establish the genuine therapeutic relationship traditionally formed between patients and physicians [16]. Several studies found that participants expressed less trust in and were less comfortable with generative AI tools than with human physicians for medical diagnosis [9,16,17]. A survey of 2039 US adults found that the majority (65.8%) reported low confidence in their healthcare system’s capability to deploy AI responsibly, and (57.7%) reported low trust that AI tools would be implemented without causing harm [17]. Accordingly, the literature supports integrating generative AI tools as a complementary adjunct to physician judgment, rather than as an independent resource, given AI’s potential to produce complex outputs that patients may find difficult to interpret [18].

The rapid, largely unregulated adoption of generative AI tools in Saudi Arabia (SA) for informal health consultations directly influences health-seeking behaviour among health consumers. Limited and insufficient studies have examined how consumers, particularly patients, respond to AI-generated health information and the impacts on their health-seeking behaviour in the Saudi context. This indicates a significant gap in the literature on the actual use and impacts of such a tool on health consumer behaviour.

Therefore, this study aims to investigate the use patterns, impact, and perspectives of generative AI tools on consumers’ health-seeking behaviours. The study’s significance lies in its capacity to advance understanding of Saudi health consumers’ behaviours beyond general attitudes toward AI tools, the technical accuracy of AI outcomes, and AI platforms’ popularity, focusing on the self-reported impacts on health-seeking behaviours. The findings are expected to benefit health care professionals in SA and worldwide by providing insight into patients’ pre-consultation expectations, informing policy development, and supporting AI developers in designing more clinically congruent tools for health consumers.

## 2. Materials and Methods

### 2.1. Study Design and Setting

The study employed a cross-sectional survey design. This design enabled the collection of quantitative data on usage patterns, impact, and perspectives on health-seeking behaviours among health consumers in SA. Data analysis identified associations between use and consumers’ characteristics and perspectives. Also, the consumers’ health-seeking behaviours and AI-based generative recommendations.

### 2.2. The Development of the Study Instrument

The study utilised a four-section, structured, and self-administered online questionnaire that was developed after a comprehensive review of the relevant literature [14,19,20,21] and discussions among the research team. These sections included (i) participants’ sociodemographic variables, (ii) health consumers’ usage patterns, (iii) impacts of the use of generative AI tools on health-seeking behaviours, and (iv) perspectives on seeking AI-based generative recommendations, which contained 10 items on a 5-point Likert Scale (1 = Strongly Disagree to 5 = Strongly Agree). The items assessed the ease of use, comprehensibility, trust in the accuracy of information, confidence in decision-making, influence on the decision to seek medical care, anxiety reduction, increased worry, comparative trust in physicians, willingness to recommend AI tools, and satisfaction. In addition, a separate multiple-response item assessing concern about using generative AI for health-related purposes was not treated as a Likert-scale item.

The questionnaire was initially developed in English specifically for this study rather than translated from a single established instrument. It was then translated into Arabic to serve the target population. Consequently, the validation process included qualitative expert review, pilot testing, and preliminary psychometric evaluation.

A panel of six experts, including three clinicians and three health informatics specialists, conducted a qualitative face and content review of the questionnaire items. All experts were fluent in both English and Arabic. The panel assessed content validity by reviewing the relevance of questionnaire items to the objectives, as well as their clarity and comprehensibility for the intended populations. No formal content validity index at the item or scale level. All modifications requested by the expert panel were completed before launching the pilot test. Appendix A provides the full questionnaire.

### 2.3. Study Population and Sampling

The study population comprised all adults (≥18 years) residing across all regions of SA. Eligibility criteria for participation were being 18 or older, having internet access, owning a digital device, and having at least one prior experience with an AI tool within the last 12 months. Adults who were below 18 years, not able to access the Internet, or did not own any digital device were not eligible to participate.

The sample size of this study was calculated using the Raosoft online calculator [22], applying a 95% confidence level, a 5% margin of error, and a conservative response distribution to provide adequate precision for proportions among eligible users. Thus, the minimum required sample size is 377 participants.

### 2.4. Data Collection

The questionnaire was distributed online via Google Forms. Recruitment was conducted in two rounds (round 1: 1 March–15 to April 2026; round 2: 20 July to 5 August 2026). The decision to reopen recruitment was made after the first descriptive/inferential analyses, to increase the sample size and improve the precision of the estimates. The questionnaire, item wording, response options, eligibility criteria, informed consent procedure, recruitment channels, and duplicate response safeguards remained unchanged. Every submission carried a date stamp, and a two-level recruitment round variable (round 1 versus round 2) was derived from it so that the two rounds could be compared and a sensitivity analysis could be run. Invitations were sent across popular social media platforms (e.g., LinkedIn, WhatsApp, and X). The invitation included a brief description of the study objective, eligibility criteria, a participant information sheet, and a direct link to the survey. Technical safeguards were implemented to prevent duplicate submissions. Participant anonymity was ensured by assigning unique study identifiers; no direct personal identifiers (e.g., name, telephone number) were collected or retained.

A pilot test was conducted with the 12 respondents who completed the online questionnaire. The pilot assessed the clarity and comprehensibility of the questions. We did not modify any items after pilot testing. Therefore, the questionnaire was administered in its original form during the main study. Eligible responses from the pilot participants were retained in the final analysis because the data collection instrument was unchanged, and the pilot responses were not analyzed as a separate sample.

Participation in this study was entirely voluntary, and no incentives were offered. Informed consent was obtained electronically at the point of survey entry. Any participant could withdraw at any time without consequences.

### 2.5. Data Analysis

After data collection, the response data were transferred to a Microsoft Excel file for initial cleaning and subsequently imported into IBM SPSS Statistics version 32 and JASP version 0.98.1. Descriptive statistics, group comparisons, reliability analyses and the logistic regression models were run in SPSS; the exploratory factor analysis was run in JASP. Descriptive statistics summarised all variables’ means and standard deviations (or medians and interquartile ranges) for continuous variables, and frequencies with percentages for categorical variables.

For the analysis of usage frequency, the five response categories were treated as ordered categories. Spearman’s rank correlation was used to examine associations between usage frequency and age, income, education, self-rated health, physician-visit frequency, and frequency of using AI before contacting a healthcare provider. The Mann–Whitney U test was used to compare usage frequency and selected Likert-item scores by gender. Characteristics and outcomes were also compared between the two recruitment rounds, using Pearson chi-square tests for categorical variables and Mann–Whitney U tests for ordinal items. Where expected cell counts fell below five, the same comparison was repeated on collapsed categories, and both results are reported. For the exploratory binary logistic regression, monthly AI use was dichotomised into frequent use (≥4 times per month; four to five times or more than five times per month) and less-frequent use (fewer than four times per month). The threshold was selected pragmatically because four or more uses per month approximates weekly use and produced adequately sized comparison groups. This was not a validated clinical threshold. The original five-category frequency variable was retained for descriptive and correlation analyses.

Predictors for the exploratory binary logistic regression were chosen in two stages. In the first stage, seven candidate variables were screened one at a time against monthly usage frequency: age, gender, education, income, self-rated health, physician-visit frequency and pre-consultation AI use. Ordinal candidates were screened with Spearman’s rank correlation and gender with the Mann–Whitney U test. The retention criterion was set in advance at *p* < 0.05. Three variables met it (age, income and pre-consultation AI use) and the other four did not (gender, education, self-rated health and physician-visit frequency). In the second stage, the three retained variables were entered simultaneously in a single block. Each was coded as an ordinal category score and modelled as a single linear term, so each odds ratio refers to a one-category increase rather than to a comparison against a reference group. No automated stepwise procedure was used at either stage, and no interaction terms were included in the main model. For transparency, the full seven-predictor model was also estimated and is reported alongside the three-predictor model in the Supplementary Materials.

Model performance was assessed with the omnibus likelihood-ratio test, the −2 log likelihood, the Cox and Snell and Nagelkerke R-squared statistics, the Hosmer–Lemeshow goodness-of-fit test and the area under the receiver operating characteristic curve. Multicollinearity was checked by re-estimating the same predictor set in a linear regression and reading the tolerance and variance inflation factor for each term, since these depend on the predictors rather than on the link function. A sensitivity analysis added recruitment round to the three-predictor model as a binary covariate, with round 1 as the reference category, to test whether the reported associations depended on when participants were recruited. A further model added round-by-predictor interaction terms to check whether the strength of any association differed between the rounds. Full model specifications, analytic sample sizes, adjusted odds ratios with 95% confidence intervals, *p*-values, collinearity diagnostics and fit statistics are given in Supplementary Materials (see Tables S4 and S5).

The psychometric properties of the 10 Likert items were evaluated among the eligible participants. Because the response options were ordinal, the exploratory factor analysis (EFA) was based on a polychoric correlation matrix, with minimum residual extraction and oblique Promax rotation. Sampling adequacy was assessed using the Kaiser–Meyer–Olkin (KMO) statistic and Bartlett’s test of sphericity. Factor retention was guided by parallel analysis based on 1000 random simulations. Internal consistency was evaluated using Cronbach’s alpha. The 10-item responses were initially examined in their recorded direction. Reverse scoring of the worry and physician-preference items changed the signs of their loadings but did not resolve their weak or ambiguous factor performance. Accordingly, no single 10-item composite score was used in the revised analysis. Instead, two factor-based subscale scores were calculated as the mean of the items with a clear primary loading on each factor. Subscale 1, usability and perceived experience, was the mean of Q20, Q21, Q25 and Q29. Subscale 2, trust and confidence, was the mean of Q22, Q23 and Q28. Three items were left out of both subscales and reported individually: Q24, which loaded on both factors, Q26, whose communality was near zero, and Q27, whose loading pattern ran counter to the construct it was written for. Internal consistency was calculated separately for each subscale, together with corrected item-total correlations and alpha-if-deleted values, and the two subscale scores were then compared with each other and across gender and recruitment round. All tests were two-sided, and *p* < 0.05 was considered statistically significant.

## 3. Results

A total of 492 participants submitted the online survey: 413 (84%) in the first round and the remaining 79 (16%) in the second round. Of these, 403 (82%) participants met the eligibility criteria (i.e., prior use of AI for health-related symptoms, information, or advice) and were included in the analytic sample. Of the 403 eligible participants, 333 (82.6%) were recruited during the first round and 70 (17.4%) in the second. In contrast, 89 (18%) participants were excluded per the eligibility criteria, including 76 (15.4%) who reported no prior use and 13 (2.6%) who were uncertain about their use. Of these excluded responses, 80 (19.4%) were excluded in the first round, and 9 (11.4%) in the second round. The analytic sample met the minimum required sample size of 377 participants. All 403 eligible participants provided complete responses to the 10 Likert items. The physician opinion item had 20 missing responses and was analysed using the 383 valid responses.

### 3.1. Demographic Findings

Among the 403 eligible participants, the largest age group was 35–44 years (26.1%). Males comprised 58.3% of the sample. The majority held a bachelor’s degree (43.9%); 16.1% held a doctoral degree. Non-healthcare employment was the most common occupational category (31.5%), while 18.4% worked in healthcare. Around 40% reported a monthly income of 20,000 SAR or above. Most participants rated their health as excellent (36.5%) or very good (40.7%), and the majority visited a physician two to three times per year (41.7%). Table 1 shows demographic characteristics in detail.

### 3.2. Usage Patterns of Generative AI Tools by Health Consumers in Saudi Arabia

Among 403 eligible participants, 197 (48.9%) reported seeking online health information via generative AI tools more than five times per month (see Table 2). The most common presenting complaints prompting action were headache (28%) followed by abdominal pain (19.4%) and joint pain (19.1%). Consultations predominantly involved moderate symptoms (43.4%), while severe and extreme symptoms accounted for 11.9% of cases. ChatGPT was the predominant platform (84.1%), followed by Gemini (38%) and DeepSeek (12.4%). The most common reasons for seeking online health information via generative AI tools were quick retrieval of health information (71.2%), timesaving (34.2%), and curiosity (30.5%). The most frequently received AI recommendation was to see a doctor soon (32.8%), followed by home management (27.5%). However, 21.1% of respondents reported not receiving clear recommendations.

### 3.3. Factors Associated with Using Generative AI Tools by Health Consumers in Saudi Arabia

Bivariate analyses revealed that the frequency of seeking online health information from generative AI tools was significantly and inversely associated with age (Spearman’s rho = −0.214, *p* < 0.001), positively associated with income (rho = 0.183, *p* < 0.001), and frequency of using generative AI tools before contacting a healthcare provider (rho = 0.384, *p* < 0.001). Usage frequency was not significantly associated with education (rho = 0.033, *p* = 0.505), self-rated health (rho = 0.088, *p* = 0.078), or physician-visit frequency (rho = 0.021, *p* = 0.671). Usage frequency did not differ significantly by gender (Mann–Whitney U = 19,105, *p* = 0.556).

In the exploratory binary logistic regression, the original five-category usage-frequency variable was dichotomised into frequent use (≥4 times per month) and less frequent use (<4 times per month). Of the seven candidate predictors screened, three met the pre-specified bivariate criterion of *p* < 0.05 and were entered simultaneously in a single block: age, household income and pre-consultation AI-use frequency. Each was modelled as an ordinal category score with a single linear term, so every odds ratio below refers to a one-category increase rather than to a comparison with a reference group. Gender, education, self-rated health and physician-visit frequency did not meet the criteria and were not entered. No stepwise procedure was used, and no interaction terms were included. The analytic sample was all 403 eligible participants, with no missing data on any model variable. Of the 403 eligible participants, 233 (57.8%) were classified as frequent users and 170 (42.2%) as less-frequent users. Each one-category increase in age was associated with lower odds of frequent use (adjusted OR = 0.699, 95% CI: 0.591–0.827, *p* < 0.001), whereas higher income was associated with higher odds (adjusted OR = 1.378, 95% CI: 1.154–1.645, *p* < 0.001). More frequent use of generative AI tools before contacting a healthcare provider was also associated with higher odds of frequent monthly use (adjusted OR = 1.895, 95% CI: 1.538–2.335, *p* < 0.001). These findings represent associations and should not be interpreted causally. The full seven-predictor model is reported alongside this one in Supplementary Materials (see Table S4). The two models agree: the four predictors that failed the screening criterion remain non-significant when forced into the model, and the three retained associations are unchanged in direction and significance.

The model was a significant improvement on the intercept-only model (omnibus χ^2^ (3) = 75.56, *p* < 0.001; −2 log likelihood = 473.23). It explained a modest share of the variance (Cox and Snell R^2^ = 0.171, Nagelkerke R^2^ = 0.230) and discriminated moderately well (area under the ROC curve = 0.752, 95% CI: 0.703–0.800). The Hosmer–Lemeshow test was non-significant, indicating acceptable calibration (χ^2^ (8) = 12.97, *p* = 0.113). Multicollinearity was not a concern: tolerance ranged from 0.912 to 0.969, and variance inflation factors ranged from 1.03 to 1.10, well within conventional limits. In the full seven-predictor model, the variance inflation factors ranged from 1.06 to 1.34. In Supplementary Materials (see Table S5) reports the fit and collinearity diagnostics for every model.

### 3.4. Impacts on Consumers’ Health-Seeking Behaviours

Table 3 presents self-reported impacts of using generative AI tools on health-seeking behaviours. Most participants took independent self-management actions (40.2%), took no action (22.8%), delayed care-seeking (22.8%), or requested immediate medical care on the same day (14.1%). Also, 22.3% of participants reported postponing a physician visit because generative AI tools reassured them, while 68% reported they had not postponed a visit and 9.7% were uncertain. 25.3% of participants reported seeking medical care because generative AI tools recommended it, whereas 64.5% did not and 10.2% were uncertain. Before contacting a healthcare provider, 27.5% of participants reported using generative AI often or almost always.

Among the 383 participants who answered the physician-opinion item, only 6.5% reported that a physician or healthcare professional encouraged them to use AI tools, 9.4% reported being warned against their use, and 17.8% reported that the professional did not comment. However, most participants (66.3%) had not told their physician or healthcare professional about their AI use.

### 3.5. Consumers’ Perspective, Satisfaction, and Concerns Toward the AI-Based Generative Recommendation

The psychometric findings did not support treating the ten Likert items as a single unidimensional composite. A two-factor exploratory solution was retained, and the two subscales are described separately below rather than summarised as one overall perspective score. The first factor, usability and perceived experience, comprised ease of use (Q20), comprehensibility (Q21), anxiety reduction (Q25) and satisfaction (Q29). This four-item subscale had a mean of 3.24 (SD = 1.00), a median of 3.25 (IQR 2.50 to 4.00) and Cronbach’s α = 0.878, with corrected item-total correlations between 0.66 and 0.78. Its mean sits just above the scale midpoint, so participants were mildly positive about how easy the tools were to use and how the experience felt. The second factor, trust and confidence, comprised trust in the accuracy of the information (Q22), confidence in acting on it (Q23) and willingness to recommend the tools to others (Q28). This three-item subscale had a mean of 2.72 (SD = 0.96), a median of 3.00 (IQR 2.00 to 3.33) and Cronbach’s α = 0.849, with corrected item-total correlations between 0.63 and 0.77. Its mean sits below the midpoint, so participants were mildly negative about trusting the content and passing it on. The two subscale scores were moderately correlated (Pearson r = 0.590, *p* < 0.001; Spearman rho = 0.594, *p* < 0.001), consistent with related but distinct constructs rather than one underlying dimension. The distance between the two means is itself the finding: the same participants found the tools easy and pleasant to use while stopping short of trusting what the tools told them. The item assessing whether using generative AI tools helped participants decide to see a doctor was retained as an individual item because it cross-loaded on both factors. The worry item (Q26) and the physician-preference item (Q27) were also excluded from both subscales, for the reasons set out below.

As shown in Table 4, the highest mean was observed for trusting physicians more than generative AI tools (Q27: M = 3.67, SD = 1.38), followed by ease of use (Q20: M = 3.37, SD = 1.22) and ease of understanding recommendations (Q21: M = 3.37, SD = 1.19). Satisfaction (Q29) had a mean of 3.17 (SD = 1.12). Lower means were observed for trust in the accuracy of AI-generated health information (Q22: M = 2.69, SD = 1.07), willingness to recommend AI tools to others (Q28: M = 2.71, SD = 1.17), and confidence in making decisions based on AI recommendations (Q23: M = 2.77, SD = 1.06).

In gender comparisons, males reported higher trust in the accuracy of AI-generated health information than females (M = 2.78 vs. 2.56; Mann–Whitney U = 17,500, *p* = 0.043). Males also reported higher satisfaction with their AI experience (M = 3.31 vs. 2.98; U = 16,320.5, *p* = 0.002). At the subscale level, the same direction held for usability and perceived experience (males M = 3.36 vs. females M = 3.08; U = 16,690, *p* = 0.008), while the difference in trust and confidence did not reach significance (M = 2.80 vs. 2.62; U = 17,552, *p* = 0.056).

The most frequently reported concerns in Q30 (see Appendix A) were that generative AI tools might provide inaccurate information (68.5%), followed by unnecessary anxiety (52.1%) and failure to detect serious problems (28.8%). Privacy concerns were reported by 22.8%, and 8.9% were concerned AI could replace physicians in the future. 13.4% of participants reported having no major concerns. Because this was a multiple-response question, the percentages were not expected to sum to 100%.

The exploratory factor analysis, based on a polychoric correlation matrix with minimum residual extraction and oblique Promax rotation, indicated adequate factorability (KMO = 0.872; Bartlett’s test of sphericity, χ^2^ (45) = 2751.14, *p* < 0.001). Parallel analysis supported retaining two factors; the first two polychoric eigenvalues were 5.31 and 1.61, while the third was 0.97 (see Table 5). The factors were moderately correlated (r = 0.55). The item “Seeking AI-based generative recommendations increased my worry” had a very low communality (0.06) and a corrected item–total correlation of 0.078; its removal would increase the raw 10-item alpha from 0.865 to 0.890, so it was reported on its own rather than included in a subscale. The physician-preference item (Q27) loaded strongly on the usability factor and negatively on the trust factor, which does not fit the construct it was written to capture. Its polychoric correlations show why: Q27 correlated between 0.47 and 0.49 with the usability items but only 0.02 to 0.04 with the two trust items, so participants who found the tools easy to use still preferred their physician, independently of how far they trusted the tools. An item that asks respondents to weigh two referents against each other in a single rating behaves unpredictably in a factor solution, so Q27 was reported separately rather than folded into either subscale or reverse-scored. These findings provide preliminary evidence for a two-dimensional structure but do not support a single global perspective score without further validation in an independent sample.

### 3.6. Comparison of the Two Recruitment Rounds and Sensitivity Analysis

Of the 403 eligible participants, 333 (82.6%) were recruited in round 1 and 70 (17.4%) in round 2. The two rounds differed on several characteristics. Round 2 participants were younger (χ^2^ (5) = 20.22, *p* = 0.001), more likely to hold a master’s degree and less likely to hold only a high school diploma (χ^2^ (5) = 23.64, *p* < 0.001), more likely to be self-employed and less likely to be retired (χ^2^ (6) = 42.60, *p* < 0.001), reported lower household income (χ^2^ (4) = 22.74, *p* < 0.001) and rated their general health less favourably (χ^2^ (4) = 13.60, *p* = 0.009). Gender (*p* = 0.099), chronic medication use (*p* = 0.908) and physician-visit frequency (*p* = 0.109) did not differ. Because some cells were sparse, age, education, occupation and self-rated health comparisons were repeated on collapsed categories and reached the same conclusions (see Supplementary Materials Table S1).

Key outcomes also differed. Frequent use, defined as four or more times per month, was reported by 210 of 333 participants (63.1%) in round 1 and 23 of 70 (32.9%) in round 2 (χ^2^ (1) = 21.64, *p* < 0.001). The five-category usage-frequency variable differed in the same direction (χ^2^ (4) = 26.56, *p* < 0.001), as did the type of recommendation received (χ^2^ (4) = 19.40, *p* < 0.001), postponing a physician visit (χ^2^ (2) = 7.48, *p* = 0.024) and the physician-opinion item (χ^2^ (3) = 16.26, *p* = 0.001). Symptom severity (*p* = 0.341), pre-consultation AI use (*p* = 0.188), the action taken afterwards (*p* = 0.237) and seeking care on AI advice (*p* = 0.099) did not differ. On the ten perspective items, round 2 participants scored higher on seven of ten, and both subscales were higher in round 2 (usability 3.54 vs. 3.18, U = 9249, *p* = 0.006; trust 3.20 vs. 2.62, U = 7466.5, *p* < 0.001). Supplementary Materials (Tables S2 and S3) present all comparisons.

In the sensitivity analysis, recruitment round was added to the three-predictor model as a binary covariate with round 1 as the reference. Round 2 was independently associated with lower odds of frequent use (adjusted OR = 0.192, 95% CI: 0.102–0.362, *p* < 0.001) and adding it improved the model on every index (−2 log likelihood 473.23 to 444.72; Nagelkerke R^2^ 0.230 to 0.306; area under the ROC curve 0.752 to 0.781; Hosmer–Lemeshow χ^2^ (8) = 6.64, *p* = 0.576). All three original predictors kept their direction and remained significant: age-adjusted OR = 0.657 (95% CI: 0.551–0.784, *p* < 0.001), income-adjusted OR = 1.269 (95% CI: 1.056–1.525, *p* = 0.011) and pre-consultation AI use-adjusted OR = 2.094 (95% CI: 1.670–2.625, *p* < 0.001). Income was affected most, its adjusted odds ratio falling from 1.378 to 1.269 and its *p*-value moving from below 0.001 to 0.011, while the age and pre-consultation associations strengthened slightly. A further model adding round-by-predictor interaction terms produced a significant block (χ^2^ (3) = 12.58, *p* = 0.006), driven by the pre-consultation term (B = 1.339, *p* = 0.010): the association between pre-consultation use and frequent use ran in the same direction in both rounds but was stronger in round 2. The substantive conclusions therefore hold after adjustment for recruitment round. The pooled estimates are nonetheless weighted towards round 1, which supplied 83% of the analytic sample, and the differences between rounds are treated as a limitation rather than as evidence that the two rounds were equivalent.

## 4. Discussion

This study investigated usage patterns, impacts, and perspectives on generative AI tools among health consumers in SA. The findings align with previous studies that found a significant positive association between high use of emerging tools in the Saudi context and younger age and greater digital fluency [2,23,24]. High usage is also positively associated with income and upcoming medical encounters, which appears to be a context-specific finding. Respondents’ perceptions varied across the dimensions of their experience with generative AI tools. The highest mean score was for trusting doctors’ recommendations over recommendations from generative AI tools (mean = 3.67). This was followed by respondents’ understanding of AI-generated recommendations and overall satisfaction with the experience (mean = 3.37 and 3.17, respectively). Overall, respondents reported moderate to high perceptions across the usability and perceived experience dimensions of generative AI tools, including ease of use, comprehensibility, anxiety reduction, and overall satisfaction. In contrast, the trust and confidence dimensions, including trust in the accuracy of AI-generated information, confidence in acting on AI-generated recommendations, and willingness to recommend these tools to others, showed lower mean scores, falling below the midpoint of the scale. This pattern suggests that although respondents generally reported favorable experiences with the usability and perceived benefits of generative AI tools, they remained relatively cautious about trusting the accuracy of the information, acting on AI-generated recommendations, and recommending these tools to others. Respondents’ concerns included receiving inaccurate health information, causing unnecessary anxiety, and breaching their privacy and safety. However, these indicators are reported separately, and this study does not provide an overall appraisal.

In this study, almost half of participants (48.9%) reported using generative AI tools more than five times a month, and ChatGPT was the most commonly used AI platform (84.1%). This descriptive finding aligns with a recent cross-sectional study investigating the real-world public use of AI chatbots for symptom checking and laboratory results in SA [23]. The study revealed that 65% of health consumers in SA used AI chatbots less than once a month to understand symptoms and lab results, while only 10% used them up to 4 times a month [23]. Thus, our findings confirmed the usage rates among Saudi adults, with more than 50% of consumers using ChatGPT more than 5 times a month to seek various types of health information. In contrast, a cross-sectional survey of 2406 American adults found that almost 40% of users accessed ChatGPT two to three times per week in 2024 [25]. Shahsavar and Choudhury (2023) examined factors influencing users’ perceptions of the decision-making process and their intention to use generative AI tools for self-diagnosis [26]. The study found that higher performance expectancy and positive risk-reward appraisals were positively associated with improved users’ perceptions of decision-making outcomes [26]. This positive perception influenced users’ intention to use the tool for self-diagnosis [26]. Observed patterns of generative AI use in the Saudi context and worldwide, as found in this study, suggest using this tool as a potential adjunct to early triage or pre-consultation information support in selected contexts. Importantly, we cannot draw conclusions about clinical integration because the scope of this cross-sectional study did not assess triage accuracy, diagnostic performance, clinical outcomes, or patient safety. Prospective studies are needed to determine whether and how generative AI tools could be used safely in future clinical pathways.

This study identified three main motivations for seeking health recommendations from generative AI tools: rapid information retrieval, time efficiency, and curiosity. Prior work showed that rapid access to information, understanding possible causes of diseases and symptoms, home management, and triage support were the principal motivations for Saudi health consumers [23].

The present study suggests that the influences of generative AI tools on health consumers’ behaviours of utilizing healthcare are limited. Most respondents reported that their decisions to visit physicians or seek urgent care were not influenced by AI-generated recommendations. However, some reported using generative AI before contacting healthcare providers and taking self-management actions afterward. This study also found that common symptoms such as headache, abdominal pain, and musculoskeletal pain were the primary triggers for seeking health information from generative AI tools. These symptoms often presented with ambiguous clinical features and vary in urgency. This suggests that generative AI was mainly used in a perceived “grey zone” of decisional uncertainty, where the health consumer was unsure whether health care was necessary or whether symptoms could be monitored or managed at home. This pattern is consistent with prior findings showing that digital self-triage tools were disproportionately used for common, non-specific presentations rather than acute emergencies [14,26].

AI tools have the potential to promote positive health behaviours for health consumers. For example, Schmieding et al. (2025) evaluated the effects of a dedicated symptom-checker mobile application on patients’ satisfaction in two emergency departments in Germany [27]. The rate of postponed physician visits in the intervention group exceeded that reported for the control group [27]. The study found no differences between the intervention and control groups in terms of patient-physician interaction or patient satisfaction [27]. One systematic review concluded that relying on digital symptom checkers could pose safety risks because of deficits in diagnostic and triage performance [28]. However, generative AI-based chatbots have been effective in supporting patient education, healthy lifestyles, smoking cessation, medication adherence, and reducing substance misuse [29].

The open-ended, dialogic nature of generative AI queries encourages health consumers to repeatedly seek information and broaden their queries. These features can amplify the prevalence of health anxiety. One systematic review reported consistent associations between AI-mediated amplification and health anxiety, internet addiction, and anxiety sensitivity [30]. In addition, most respondents preferred not to disclose health recommendations received from generative AI tools to their physicians or healthcare professionals, although such disclosure may facilitate earlier recognition of serious conditions when AI tools prompt urgent care-seeking. Prior research suggested that a non-disclosure pattern indicated patients’ negative view of obtaining health information from AI, fearing it may harm their relationships with physicians or lead to disapproval or distrust from them [31]. Therefore, health consumers need appropriate health education initiatives on sharing protected health information in digital health media to increase trust and transparency [32]. Importantly, healthcare providers need to proactively adapt to the major shift in consumers’ health behaviors. Providers should encourage patients to share AI-generated information to clarify misconceptions or correct misinformation.

Importantly, the study reveals a coexistence of high utilisation with persistently low trust, a pattern that can be described as tension between perceiving the usefulness of generative AI tools and trusting their recommendations. In this study, trust in the diagnostic accuracy of generative AI tools was low (mean = 2.69, SD = 1.07). This divergence between use frequency and trust aligns with Saudi-specific research on generative AI tools in health care. Alshehri et al. (2026) reported low trust, with most respondents expressing low or moderate levels [23]. However, Alharbi et al. (2026) observed moderate-to-high trust with notable variability and lower trust among older users and those with strong relationships with their physicians [24]. The trust deficit observed in this study is consistent with multinational evidence from a large-scale cross-sectional survey of 13,806 hospital patients across 43 countries [31], which found that fewer than half of respondents expressed positive attitudes across all trust-related items. This study offers evidence of the sample’s awareness of the risk of receiving hallucinations as inaccurate health recommendations from AI tools. This aligns with global evidence indicating that about 70% of AI users in clinical contexts were concerned about the accuracy of health information obtained [24].

This study has several limitations that constrain its conclusions. First, the cross-sectional design does not support causal inference. Therefore, reports of delayed care-seeking or self-treatment should not be interpreted as causal effects of generative AI use. Second, the sample’s demographic characteristics limit the findings’ generalizability. Most participants had relatively high educational attainment and household income. Consequently, the findings may not generalize to lower-resource populations or adults with limited digital access. Third, although the same questionnaire and recruitment procedures were used in the two recruitment periods, residual recruitment or selection bias cannot be excluded. Thus, findings should be interpreted cautiously and should not be assumed to represent all adults residing in SA. Fourth, excluding adults without previous experience using generative AI tools limits the generalizability of the findings to the broader adult population, including individuals who have not yet used these tools. In addition, participants’ actual use of generative AI could not be independently verified. Reliance on self-reported use may therefore have introduced misclassification, recall bias, and social desirability bias, particularly concerning the frequency of AI use and behavioural changes following AI consultations. Finally, dichotomising the original five-category usage-frequency variable into frequent and less frequent use may have resulted in a loss of information and obscured differences between adjacent usage categories. Accordingly, the regression findings should be regarded as exploratory, and the selected threshold should not be considered a definitive definition of frequent generative AI use.

## 5. Conclusions

This study provided evidence on the use, impacts, and motivations of a sample of Saudi health consumers seeking health recommendations from generative AI tools. High usage was associated with younger age, higher income, and greater pre-consultation use. Motivations included rapid information retrieval, time efficiency, and curiosity. Findings showed tension between perceiving the usefulness of seeking recommendations from generative AI tools and trusting their impact on patient safety, due to inaccurate information and the omission of serious diagnostic issues. Most participants reported not disclosing their use to physicians. Future work needs close collaboration among healthcare providers, AI developers, health-technology vendors, and regulators to promote trustworthy, transparent AI tools and encourage patients to discuss AI-generated health information during clinical encounters. Finally, this study did not assess adverse clinical outcomes and cannot generalize to all health consumers in Saudi Arabia or similar health contexts because of the study limitations.

## Figures and Tables

**Table 1:** Participants’ Demographic Information (N = 403).

Variable	Sub-Variable	Frequency	Percent
Q1: Age	18–24 years	88	21.8
	25–34 years	80	19.9
	35–44 years	105	26.1
	45–54 years	69	17.1
	55–64 years	53	13.2
	65 years or older	8	2.0
Q2: Gender	Male	235	58.3
	Female	168	41.7
Q3: Education	Less than high school	5	1.2
	High school diploma or equivalent	74	18.4
	Diploma (post-secondary)	29	7.2
	Bachelor’s degree	177	43.9
	Master’s degree	53	13.2
	Doctoral degree (PhD/MD)	65	16.1
Q4: Occupation	Student	82	20.3
	Employed in healthcare	74	18.4
	Employed in non-healthcare	127	31.5
	Self-employed	23	5.7
	Unemployed/looking for work	16	4.0
	Housewife	33	8.2
	Retired	48	11.9
Q5: Monthly income	Less than 5000 SAR	29	7.2
	5000–9999 SAR	50	12.4
	10,000–14,999 SAR	85	21.1
	15,000–19,999 SAR	77	19.1
	20,000 SAR or more	162	40.2
Q6: Self-perception of general health	Excellent	147	36.5
	Very Good	164	40.7
	Good	80	19.9
	Fair	11	2.7
	Poor	1	0.2
Q7: Taking chronic medications	Yes	153	38.0
	No	250	62.0
Q8: Frequency of visiting doctors per year	Once a year or less	128	31.8
	2–3 times a year	168	41.7
	4–6 times a year	78	19.4
	More than 6 times a year	29	7.2

**Table 2:** Health Consumers’ Usage Pattern of Generative Artificial Intelligence Tools (N = 403).

Usage Pattern	Measure	Frequency	Percent
Q9: Frequency of using generative AI tools per month for seeking online health information	Less than once a month	49	12.2
	Once a month	44	10.9
	2–3 times a month	77	19.1
	4–5 times a month	36	8.9
	More than 5 times a month	197	48.9
Q10: The most generative AI tool was mostly used to seek health online information and recommendation *	ChatGPT	339	84.1
	Grok	31	7.7
	Gemini	153	38.0
	Claude	30	7.4
	DeepSeek	50	12.4
	Manus	2	0.5
	Perplexity	13	3.2
	Poe	6	1.5
	Microsoft Copilot	26	6.5
	Others	8	2.0
	Not sure	15	3.7
Q11: The main complaints prompting seeking online health information from generative AI tools *	Headache	113	28.0
	Abdominal Pain	78	19.4
	Joint & muscle pain	77	19.1
	Skin problem	49	12.2
	Palpitation	55	13.6
	Cough	54	13.4
	Dyspnea	47	11.7
	Chest pain	48	11.9
	Dysuria	37	9.2
	Others	94	23.3
Q12: The main purposes to seek online health information from generative AI tools *	To seek immediate health information	287	71.2
	To determine the need for urgent care	105	26.1
	To prepare for a doctor’s visit	80	19.9
	To save time	138	34.2
	To save money	68	16.9
	Out of curiosity about generative AI	123	30.5
	Difficulty accessing healthcare	38	9.4
	Others	8	2.0
Q13: Self-reported assessment of severity of health symptoms before seeking online health information from AI tools	Very mild	61	15.1
	Mild	119	29.5
	Moderate	175	43.4
	Severe	40	9.9
	Extreme	8	2.0
Q14: Type of health recommendation obtained by generative AI tools	Manage symptoms at home	111	27.5
	See a doctor soon	132	32.8
	Go to emergency care	19	4.7
	No clear recommendation	85	21.1
	I do not remember	56	13.9
Q15: Frequency of seeking online health information from AI tools before a medical encounter	Almost never	49	12.2
	Rarely	94	23.3
	Sometimes	149	37.0
	Often	65	16.1
	Almost always	46	11.4

* Multiple Response Items. Percentages are based on N = 403; therefore, the sum of percentages may exceed 100%. The “Others” category for Q10, Q11 and Q12 comprises free-text responses that did not match any listed option.

**Table 3:** Impacts of Using Generative Artificial Intelligence Tools on Health-Seeking Behaviours (N = 403).

Impact	Sub-Variable	Frequency	Percent
Q16: Taking consecutive actions after receiving recommendations from generative AI tools	Sought medical care at the same day	57	14.1
	Delayed but eventually sought care	92	22.8
	Managed the issue myself	162	40.2
	Did not do anything/ignored it	92	22.8
Q17: Postponing doctor visits based on AI recommendations	Yes	90	22.3
	No	274	68.0
	Not sure	39	9.7
Q18: Seeking urgent care based on health recommendations obtained from generative AI tools	Yes	102	25.3
	No	260	64.5
	Not sure	41	10.2
Q19: Physicians’ recommendation after using generative AI tools for health purposes *	Encouraged me	25	6.5
	Warned me	36	9.4
	Did not comment	68	17.8
	I did not tell him/her	254	66.3

* The physicians’ opinion item received 383 valid responses, where 20 responses were missing.

**Table 4:** Health Consumers’ Perspectives toward the Use of Generative Artificial Intelligence Tools (N = 403).

Item	Strongly Disagree (%)	Disagree (%)	Neutral (%)	Agree (%)	Strongly Agree (%)	Mean (SD) *
Q20: Seeking online health information from generative AI tools was easy to use	39 (9.7)	58 (14.4)	100 (24.8)	127 (31.5)	79 (19.6)	3.37 (1.22)
Q21: I understand recommendations obtained by generative AI tools	36 (8.9)	61 (15.1)	92 (22.8)	146 (36.2)	68 (16.9)	3.37 (1.19)
Q22: I trusted the accuracy of health information obtained from generative AI tools	67 (16.6)	95 (23.6)	152 (37.7)	74 (18.4)	15 (3.7)	2.69 (1.07)
Q23: I felt confident making decisions based on AI-based generative recommendations	59 (14.6)	96 (23.8)	140 (34.7)	96 (23.8)	12 (3.0)	2.77 (1.06)
Q24: Generative AI tools helped me decide to see a doctor	43 (10.7)	86 (21.3)	115 (28.5)	124 (30.8)	35 (8.7)	3.05 (1.14)
Q25: Seeking AI-based generative recommendations reduced my anxiety about health	44 (10.9)	89 (22.1)	113 (28.0)	114 (28.3)	43 (10.7)	3.06 (1.17)
Q26: Seeking AI-based generative recommendations increased my worry	57 (14.1)	96 (23.8)	120 (29.8)	100 (24.8)	30 (7.4)	2.88 (1.16)
Q27: I trusted doctors more than generative AI tools for health advice	48 (11.9)	41 (10.2)	61 (15.1)	99 (24.6)	154 (38.2)	3.67 (1.38)
Q28: I would recommend generative AI tools to others	79 (19.6)	88 (21.8)	127 (31.5)	87 (21.6)	22 (5.5)	2.71 (1.17)
Q29: Overall, I was satisfied with my experience with generative AI tools	38 (9.4)	67 (16.6)	125 (31.0)	133 (33.0)	40 (9.9)	3.17 (1.12)

* The mean of the item is based on the original 5-point Likert scale.

**Table 5:** Exploratory Factor Analysis of the 10-item perspective Scale.

Item	Factor 1: Usability/Experience	Factor 2: Trust/Confidence	Communality
Q20: Seeking online health information from generative AI tools was easy to use	0.76	0.13	0.69
Q21: I understand recommendations obtained by generative AI tools	0.73	0.20	0.74
Q22: I trusted the accuracy of health information obtained from generative AI tools	−0.06	0.95	0.84
Q23: I felt confident making decisions based on AI-based generative recommendations	−0.04	0.91	0.78
Q24: Generative AI tools helped me decide to see a doctor	0.43	0.46	0.62
Q25: Seeking AI-based generative recommendations reduced my anxiety about health	0.56	0.28	0.57
Q26: Seeking AI-based generative recommendations increased my worry	0.29	−0.23	0.06
Q27: I trusted doctors more than generative AI tools for health advice	0.94	−0.47	0.62
Q28: I would recommend generative AI tools to others	0.03	0.73	0.55
Q29: Overall, I was satisfied with my experience with generative AI tools	0.62	0.36	0.76

Note. Extraction was based on a polychoric correlation matrix with minimum residual estimation and oblique Promax rotation (N = 403). Values in the first two columns are pattern coefficients; the two factors were correlated at r = 0.55. Sampling adequacy was acceptable (KMO = 0.872; Bartlett’s test of sphericity χ^2^ (45) = 2751.14, p < 0.001), and parallel analysis based on 1000 simulations retained two factors (eigenvalues 5.31, 1.61 and 0.97). Q20, Q21, Q25 and Q29 form the usability and perceived experience subscale (α = 0.878); Q22, Q23 and Q28 form the trust and confidence subscale (α = 0.849). Q24 cross-loaded, Q26 had a communality of 0.06, and Q27 loaded in a direction inconsistent with its content, so all three are reported as individual items and belong to neither subscale.

## Data Availability

The datasets presented in this article are not readily available because the data are restricted due to institutional regulations related to health data privacy. Requests to access these datasets should be directed to the King Abdullah International Medical Research Center (KAIMRC).

## Supplementary Materials

The following supporting information can be downloaded at: https://www.mdpi.com/article/10.3390/healthcare14213558/s1. Table S1: Demographic characteristics by recruitment round (N = 403); Table S2: Usage patterns and health-seeking outcomes by recruitment round (N = 403); Table S3: Perspective items and subscale scores by recruitment round (N = 403); Table S4: Binary logistic regression models predicting frequent use of generative AI tools (≥4 times per month); Table S5: Model fit and multicollinearity diagnostics.

## Appendix A. A Cross-Sectional Survey

Section I: Demographic Information.

**No.**	**Variable**	**Sub-Variable**
Q1	Age	18–24 years
		25–34 years
		35–44 years
		45–54 years
		55–64 years
		65 years or older
Q2	Gender	Male
		Female
Q3	Education	Less than high school
		High school diploma or equivalent
		Diploma (post-secondary)
		Bachelor’s degree
		Master’s degree
		Doctoral degree (PhD/MD)
Q4	Occupation	Student
		Employed in healthcare
		Employed in non-healthcare
		Self-employed
		Unemployed/looking for work
		Housewife
		Retired
Q5	Monthly income	Less than 5000 SAR
		5000–9999 SAR
		10,000–14,999 SAR
		15,000–19,999 SAR
		20,000 SAR or more
Q6	Self-perception of general health	Excellent
		Very Good
		Good
		Fair
		Poor
Q7	Taking chronic medications	Yes
		No
Q8	Frequency of visiting doctors per year	Once a year or less
		2–3 times a year
		4–6 times a year
		More than 6 times a year

Section II: Usage Patterns.

**No.**	**Usage Pattern**	**Measure**
Q9	Frequency of using generative AI tools per month for seeking online health information	Less than once a month
		Once a month
		2–3 times a month
		4–5 times a month
		More than 5 times a month
Q10	The most generative AI tool was mostly used to seek health online information and recommendation	ChatGPT
		Grok
		Gemini
		Claude
		DeepSeek
		Manus
		Perplexity
		Poe
		Microsoft Copilot
		Others
		Not sure
Q11	The main complaints prompting seeking online health information from generative AI tools. (Choose all options).	Headache
		Abdominal Pain
		Joint & muscle pain
		Skin problem
		Palpitation
		Cough
		Dyspnea
		Chest pain
		Dysuria
		Others
Q12	The main purposes to seek online health information from generative AI tools	To seek immediate health information
		To determine the need for urgent care
		To prepare for a doctor’s visit
		To save time
		To save money
		Out of curiosity about generative AI
		Difficulty accessing healthcare
		Others
Q13	Self-reported assessment of severity of health symptoms before seeking online health information from AI tools	Very mild
		Mild
		Moderate
		Severe
		Extreme
Q14	Type of health recommendation obtained by generative AI tools	Manage symptoms at home
		See a doctor soon
		Go to emergency care
		No clear recommendation
		I do not remember
Q15	Frequency of seeking online health information from AI tools before a medical encounter	Almost never
		Rarely
		Sometimes
		Often
		Almost always

Section III: Impacts on Health-Seeking Behaviour.

**No.**	**Impact**	**Sub-Variable**
Q16	Taking consecutive actions after receiving recommendations from generative AI tools	Sought medical care at the same day
		Delayed but eventually sought care
		Managed the issue myself
		Did not do anything/ignored it
Q17	Postponing doctor visits based on AI recommendations	Yes
		No
		Not sure
Q18	Seeking urgent care based on health recommendations obtained from generative AI tools	Yes
		No
		Not sure
Q19	Physicians’ recommendation after using generative AI tools for health purposes	Encouraged me
		Warned me
		Did not comment
		I did not tell him/her

Section VI: Health Consumers’ Perspectives.

**No.**	**Item**	**Strongly** **Disagree (%)**	**Disagree** **(%)**	**Neutral** **(%)**	**Agree** **(%)**		**Strongly** **Agree** **(%)**
Q20	Seeking online health information from generative AI tools was easy to use	^--^	^--^	^--^	^--^		^--^
Q21	I understand recommendations obtained by generative AI tools	^--^	^--^	^--^	^--^		^--^
Q22	I trusted the accuracy of health information obtained from generative AI tools	^--^	^--^	^--^	^--^		^--^
Q23	I felt confident making decisions based on AI-based generative recommendations	^--^	^--^	^--^	^--^		^--^
Q24	Generative AI tools helped me decide to see a doctor	^--^	^--^	^--^	^--^		^--^
Q25	Seeking AI-based generative recommendations reduced my anxiety about health	^--^	^--^	^--^	^--^		^--^
Q26	Seeking AI-based generative recommendations increased my worry	^--^	^--^	^--^	^--^		^--^
Q27	I trusted doctors more than generative AI tools for health advice	^--^	^--^	^--^	^--^		^--^
Q28	I would recommend generative AI tools to others	^--^	^--^	^--^	^--^		^--^
Q29	Overall, I was satisfied with my experience with generative AI tools						
Q30	Concerns about seeking AI-based generative recommendations	They may provide inaccurate information			Yes	No	
		They might miss serious issues			Yes	No	
		They could cause unnecessary anxiety			Yes	No	
		My privacy/data may not be safe			Yes	No	
		They could replace doctors in the future			Yes	No	
		I have no major concerns			Yes	No	
		Others			Yes	No	

## References

[1] Huisman M., Joye S., Biltereyst D. (2020). Searching for health: Doctor Google and the shifting dynamics of the middle-aged and older adult patient-physician relationship and interaction. J. Aging Health.

[2] Wong D.K.K., Cheung M.K. (2019). Online health information seeking and eHealth literacy among patients attending a primary care clinic in Hong Kong: A cross-sectional survey. J. Med. Internet Res..

[3] Wang C., Wang F., Li S., Ren Q.-W., Tan X., Fu Y., Liu D., Qian G., Cao Y., Yin R. (2025). Patient triage and guidance in emergency departments using large language models: Multimetric study. J. Med. Internet Res..

[4] Li J., Theng Y.L., Foo S. (2016). Predictors of online health information seeking behavior: Changes between 2002 and 2012. Health Inform. J..

[5] Iqbal U., Tanweer A., Rahmanti A.R., Greenfield D., Lee L.T.-J., Li Y.-C.J. (2025). Impact of large language model (ChatGPT) in healthcare: An umbrella review and evidence synthesis. J. Biomed. Sci..

[6] Solano C., Tarazona N., Angarita G.P., Medina A.A., Ruiz S., Pedroza V.M., Traxer O. (2024). ChatGPT in urology: Bridging knowledge and practice for tomorrow’s healthcare, a comprehensive review. J. Endourol..

[7] Yun H.S., Bickmore T. (2025). Online health information-seeking in the era of large language models: Cross-sectional web-based survey study. J. Med. Internet Res..

[8] Luo A., Qin L., Yuan Y., Yang Z., Liu F., Huang P., Xie W. (2022). The effect of online health information seeking on physician-patient relationships: Systematic review. J. Med. Internet Res..

[9] Juravle G., Boudouraki A., Terziyska M., Rezlescu C. (2020). Trust in artificial intelligence for medical diagnoses. Prog. Brain Res..

[10] Wu D., Lowry P.B., Zhang D., Tao Y. (2022). Patient trust in physicians matters-understanding the role of a mobile patient education system and patient-physician communication in improving patient adherence behavior: Field study. J. Med. Internet Res..

[11] Wu Q., Jin Z., Wang P. (2021). The relationship between the physician-patient relationship, physician empathy, and patient trust. J. Gen. Intern Med..

[12] Schenkel S.K., Jungmann S.M., Gropalis M., Witthöft M. (2021). Conceptualizations of cyberchondria and relations to the anxiety spectrum: Systematic review and meta-analysis. J. Med. Internet Res..

[13] McMullan R.D., Berle D., Arnáez S., Starcevic V. (2019). The relationships between health anxiety, online health information seeking, and cyberchondria: Systematic review and meta-analysis. J. Affect. Disord..

[14] Meyer A.N.D., Giardina T.D., Spitzmueller C., Shahid U., Scott T.M.T., Singh H. (2020). Patient perspectives on the usefulness of an artificial intelligence-assisted symptom checker: Cross-sectional survey study. J. Med. Internet Res..

[15] Levine D.M., Tuwani R., Kompa B., Varma A., Finlayson S.G., Mehrotra A., Beam A. (2023). The diagnostic and triage accuracy of the GPT-3 artificial intelligence model. medRxiv.

[16] Hatherley J.J. (2020). Limits of trust in medical AI. J. Med. Ethics.

[17] Nong P., Platt J. (2025). Patients’ trust in health systems to use artificial intelligence. JAMA Netw. Open.

[18] Lainjo B. (2024). Integrating artificial intelligence into healthcare systems: Opportunities and challenges. Acad. Med..

[19] Carmona K.A., Chittamuru D., Kravitz R.L., Ramondt S., Ramírez A.S. (2022). Health information seeking from an intelligent web-based symptom checker: Cross-sectional questionnaire study. J. Med. Internet Res..

[20] Mahlknecht A., Engl A., Piccoliori G., Wiedermann C.J. (2023). Supporting primary care through symptom checking artificial intelligence: A study of patient and physician attitudes in Italian general practice. BMC Prim. Care.

[21] E Morse K., Ostberg N.P., Jones V.G., Chan A.S. (2020). Use characteristics and triage acuity of a digital symptom checker in a large integrated health system: Population-based descriptive study. J. Med. Internet Res..

[22] Raosoft Inc. Sample Size Calculator. https://raosoftcalculator.com/.

[23] Alshehri N.A., Aljohani S., Alruwaili S.A., Binsalamh L.F., Alshehri T.A., Aljammaz J.A., Alrafee A.M. (2026). Public use of AI chatbots for symptom and laboratory interpretation and risky health behavior: A cross-sectional study in Saudi Arabia. Int. J. Med. Dev. Ctries..

[24] Alharbi B.S., Aljabri M.M., Ali E.A. (2026). Determinants of trust in artificial intelligence (AI) for health-related decision-making among adults in Saudi Arabia: A cross-sectional study. Healthcare.

[25] Ayo-Ajibola O., Davis R.J., E Lin M., Riddell J., Kravitz R.L. (2024). Characterizing the adoption and experiences of users of artificial intelligence-generated health information in the United States: Cross-sectional questionnaire study. J. Med. Internet Res..

[26] Shahsavar Y., Choudhury A. (2023). User intentions to use ChatGPT for self-diagnosis and health-related purposes: Cross-sectional survey study. JMIR Hum. Factors.

[27] Schmieding M.L., Kopka M., Bolanaki M., Napierala H., Altendorf M.B., Kuschick D., Piper S.K., Scatturin L., Schmidt K., Schorr C. (2025). Impact of a symptom checker app on patient-physician interaction among self-referred walk-in patients in the emergency department: Multicenter, parallel-group, randomized, controlled trial. J. Med. Internet Res..

[28] Wallace W., Chan C., Chidambaram S., Hanna L., Iqbal F.M., Acharya A., Normahani P., Ashrafian H., Markar S.R., Sounderajah V. (2022). The diagnostic and triage accuracy of digital and online symptom checker tools: A systematic review. npj Digit. Med..

[29] Aggarwal A., Tam C.C., Wu D., Li X., Qiao S. (2023). Artificial intelligence-based chatbots for promoting health behavioral changes: Systematic review. J. Med. Internet Res..

[30] Miezah D., Adzakpa R., Bani E.M.A., Obeng P. (2026). Prevalence and associated factors of cyberchondria: A scoping review. Sci. World J..

[31] Busch F., Hoffmann L., Xu L., Zhang L.J., Hu B., García-Juárez I., Toapanta-Yanchapaxi L.N., Gorelik N., Gorelik V., Rodriguez-Granillo G.A. (2025). Multinational attitudes toward AI in health care and diagnostics among hospital patients. JAMA Netw. Open.

[32] Al-Somali S.A. (2025). Integrating artificial intelligence (AI) in healthcare: Advancing older adults’ health management in Saudi Arabia through AI-powered chatbots. PeerJ Comput. Sci..

