# Supplementary material for: Assessing the Use, Impact, and Perspectives of Generative Artificial Intelligence Among Health Consumers: A Cross-Sectional Survey in Saudi Arabia
Source: Healthcare (Basel). 2026 Oct 22;14(21):3558. doi: 10.3390/healthcare14213558 (PMC13762718; doi:10.3390/healthcare14213558)
Supplement: Supplementary file 1 [file healthcare-14-03558-s001.zip › healthcare-4434832-supplementary.pdf]

Supplementary materials

The analytic sample is the 403 eligible participants: 333 in round 1 (1 March to 15 April 2026) and 70 in round 2 (20 July to 5 August 2026).

Table S1. Demographic characteristics by recruitment round (N = 403).

Characteristic	Category	Round 1 (n = 333) n (%)	Round 2 (n = 70) n (%)	χ^2 (df)	p
**Age**	18-24 years	75 (22.5)	13 (18.6)	20.22 (5)	.001
	25-34 years	55 (16.5)	25 (35.7)		
	35-44 years	84 (25.2)	21 (30.0)		
	45-54 years	61 (18.3)	8 (11.4)		
	55-64 years	51 (15.3)	2 (2.9)		
	65 years or older	7 (2.1)	1 (1.4)		
**Gender**	Male	188 (56.5)	47 (67.1)	2.72 (1)	.099
	Female	145 (43.5)	23 (32.9)		
**Education**	Less than high school	5 (1.5)	0 (0.0)	23.64 (5)	<.001
	High school diploma or equivalent	71 (21.3)	3 (4.3)		
	Diploma (post-secondary)	22 (6.6)	7 (10.0)		
	Bachelor's degree	140 (42.0)	37 (52.9)		
	Master's degree	36 (10.8)	17 (24.3)		
	Doctoral degree (PhD/MD)	59 (17.7)	6 (8.6)		
**Occupation**	Student	68 (20.4)	14 (20.0)	42.60 (6)	<.001
	Employed in healthcare	65 (19.5)	9 (12.9)		
	Employed in non-healthcare	105 (31.5)	22 (31.4)		
	Self-employed	8 (2.4)	15 (21.4)		

	Unemployed / looking for work	14 (4.2)	2 (2.9)		
	Housewife	28 (8.4)	5 (7.1)		
	Retired	45 (13.5)	3 (4.3)		
**Monthly income**	Less than 5,000 SAR	16 (4.8)	13 (18.6)	22.74 (4)	<.001
	5,000-9,999 SAR	39 (11.7)	11 (15.7)		
	10,000-14,999 SAR	68 (20.4)	17 (24.3)		
	15,000-19,999 SAR	64 (19.2)	13 (18.6)		
	20,000 SAR or more	146 (43.8)	16 (22.9)		
**Self-rated health**	Excellent	129 (38.7)	18 (25.7)	13.60 (4)	.009
	Very good	138 (41.4)	26 (37.1)		
	Good	59 (17.7)	21 (30.0)		
	Fair	6 (1.8)	5 (7.1)		
	Poor	1 (0.3)	0 (0.0)		
**Chronic medication**	Yes	126 (37.8)	27 (38.6)	0.01 (1)	.908
	No	207 (62.2)	43 (61.4)		
**Doctor visits per year**	Once a year or less	104 (31.2)	24 (34.3)	6.05 (3)	.109
	2-3 times a year	141 (42.3)	27 (38.6)		
	4-6 times a year	60 (18.0)	18 (25.7)		
	More than 6 times a year	28 (8.4)	1 (1.4)		

Note. *Pearson chi-square tests. Minimum expected cell counts: age 1.39, education 0.87, occupation 2.78, self-rated health 0.17. Because these fall below five, the four comparisons were repeated on collapsed categories, with the same conclusions: age in four categories χ^2 (3) = 18.85, p < .001; education in three categories χ^2 (2) = 12.79, p = .002; occupation in five categories χ^2 (4) = 41.46, p < .001; self-rated health in three categories χ^2 (2) = 11.58, p = .003. Income in three categories gave χ^2 (2) = 15.80, p < .001*

Table S2. Usage patterns and health-seeking outcomes by recruitment round (N = 403).

**Variable**	**Category**	**Round 1 (n = 333) n (%)**	**Round 2 (n = 70) n (%)**	**χ^2 (df)**	**p**
**AI use frequency per month**	Less than once a month	38 (11.4)	11 (15.7)	26.56 (4)	<.001
	Once a month	29 (8.7)	15 (21.4)		

	2-3 times a month	56 (16.8)	21 (30.0)		
	4-5 times a month	29 (8.7)	7 (10.0)		
	More than 5 times a month	181 (54.4)	16 (22.9)		
**Frequent use (4+ per month)**	Frequent use (4 or more per month)	210 (63.1)	23 (32.9)	21.64 (1)	<.001
	Less frequent use (fewer than 4 per month)	123 (36.9)	47 (67.1)		
**Symptom severity**	Very mild	54 (16.2)	7 (10.0)	4.51 (4)	.341
	Mild	99 (29.7)	20 (28.6)		
	Moderate	144 (43.2)	31 (44.3)		
	Severe	31 (9.3)	9 (12.9)		
	Extreme	5 (1.5)	3 (4.3)		
**Recommendation received**	Manage symptoms at home	82 (24.6)	29 (41.4)	19.40 (4)	<.001
	See a doctor soon	107 (32.1)	25 (35.7)		
	Go to emergency care	13 (3.9)	6 (8.6)		
	No clear recommendation	78 (23.4)	7 (10.0)		
	I do not remember	53 (15.9)	3 (4.3)		
**Pre-consultation AI use**	Almost never	42 (12.6)	7 (10.0)	6.15 (4)	.188
	Rarely	78 (23.4)	16 (22.9)		
	Sometimes	126 (37.8)	23 (32.9)		
	Often	47 (14.1)	18 (25.7)		
	Almost always	40 (12.0)	6 (8.6)		
**Action taken**	Sought medical care the same day	44 (13.2)	13 (18.6)	4.23 (3)	.237
	Delayed but eventually sought care	76 (22.8)	16 (22.9)		
	Managed the issue myself	131 (39.3)	31 (44.3)		

	Did not do anything / ignored it	82 (24.6)	10 (14.3)		
**Postponed a visit**	Yes	70 (21.0)	20 (28.6)	7.48 (2)	.024
	No	225 (67.6)	49 (70.0)		
	Not sure	38 (11.4)	1 (1.4)		
**Sought care on AI advice**	Yes	78 (23.4)	24 (34.3)	4.62 (2)	.099
	No	218 (65.5)	42 (60.0)		
	Not sure	37 (11.1)	4 (5.7)		
**Physician's response**	Encouraged me	14 (4.5)	11 (15.7)	16.26 (3)	.001
	Warned me	27 (8.6)	9 (12.9)		
	Did not comment	53 (16.9)	15 (21.4)		
	I did not tell him/her	219 (70.0)	35 (50.0)		

Note. *Pearson chi-square tests. The physician-opinion item was answered by 313 participants in round 1 and 70 in round 2; its percentages use those denominators. Ordinal comparisons of the same variables gave: usage frequency U = 7,834, p <.001; pre-consultation use U = 10,929, p = .396; symptom severity U = 10,272, p = .098*

Table S3. Perspective items and subscale scores by recruitment round (N = 403).

Item	Round 1 M (SD)	Round 2 M (SD)	Mann-Whitney U	p
**Q20 Easy to use**	3.32 (1.24)	3.59 (1.12)	10,300	.115
**Q21 Recommendations understandable**	3.28 (1.19)	3.79 (1.10)	8,848.5	.001
**Q22 Trusted the accuracy**	2.62 (1.09)	3.03 (0.88)	9,157	.003
**Q23 Confident deciding**	2.66 (1.08)	3.26 (0.85)	8,037.5	<.001
**Q24 Helped decide to see a doctor**	2.95 (1.13)	3.54 (1.03)	8,171	<.001
**Q25 Reduced my anxiety**	3.00 (1.18)	3.31 (1.11)	9,991.5	.053
**Q26 Increased my worry**	2.95 (1.14)	2.50 (1.16)	9,087	.003
**Q27 Trusted doctors more**	3.60 (1.43)	3.99 (1.10)	10,281	.107
**Q28 Would recommend**	2.59 (1.16)	3.31 (1.02)	7,499	<.001
**Q29 Satisfied overall**	3.11 (1.13)	3.46 (0.97)	9,450.5	.010
**Subscale 1: usability / experience**	3.18 (1.02)	3.54 (0.90)	9,249	.006

**Subscale 2: trust / confidence**	2.62 (0.97)	3.20 (0.81)	7,466.5	<.001

Note. *Mann-Whitney U tests. Items are scored 1 (strongly disagree) to 5 (strongly agree). Subscale 1 is the mean of Q20, Q21, Q25 and Q29; subscale 2 is the mean of Q22, Q23 and Q28*

Table S4. Binary logistic regression models predicting frequent use of generative AI tools (≥ 4 times per month).

Model and predictor	B	SE	Wald	Adjusted OR	95% CI	p
Model 1: three retained predictors						
**Age**	−0.358	0.086	17.47	0.699	0.591–0.827	<.001
**Household income**	0.321	0.090	12.58	1.378	1.154–1.645	<.001
**Pre-consultation AI use**	0.639	0.107	35.99	1.895	1.538–2.335	<.001
**Constant**	−1.650	0.454	13.24	0.192	—	<.001
Model 2: all seven candidate predictors						
**Age**	−0.406	0.096	17.91	0.666	0.552–0.804	<.001
**Gender (male vs female)**	−0.228	0.235	0.94	0.796	0.502–1.261	.331
**Education**	0.016	0.098	0.03	1.017	0.840–1.231	.866
**Household income**	0.347	0.098	12.67	1.415	1.169–1.713	<.001
**Self-rated health**	0.081	0.150	0.29	1.084	0.807–1.455	.592
**Physician visits per year**	0.226	0.146	2.40	1.254	0.942–1.669	.121
**Pre-consultation AI use**	0.622	0.108	33.28	1.862	1.508–2.300	<.001
**Constant**	−2.275	0.910	6.26	0.103	—	.012
Model 3: Model 1 plus recruitment round						
**Age**	−0.420	0.090	21.73	0.657	0.551–0.784	<.001
**Household income**	0.238	0.094	6.48	1.269	1.056–1.525	.011
**Pre-consultation AI use**	0.739	0.115	40.96	2.094	1.670–2.625	<.001
**Recruitment round 2 (vs round 1)**	−1.651	0.324	25.95	0.192	0.102–0.362	<.001
**Constant**	−1.144	0.476	5.77	0.319	—	.016

Note. *Outcome: frequent use, coded 1 for four or more times per month and 0 for fewer than four. Analytic n = 403 for all three models, with no missing data on any model variable; 233 participants (57.8%) were frequent users. Age, income, education, self-rated health, physician-visit frequency and pre-consultation AI use were entered as ordinal*

category scores with a single linear term, so each adjusted odds ratio refers to a one-category increase. Gender and recruitment round were entered as binary indicators, with female and round 1 as the reference categories. All predictors were entered simultaneously in a single block; no stepwise selection was used

Table S5. Model fit and multicollinearity diagnostics.

**Statistic**	**Model 1**	**Model 2**	**Model 3**
**−2 log likelihood**	473.23	469.22	444.72
**Omnibus χ^2 (df)**	75.56 (3)	79.57 (7)	104.07 (4)
**Omnibus p**	<.001	<.001	<.001
**Cox and Snell R^2**	0.171	0.179	0.228
**Nagelkerke R^2**	0.230	0.241	0.306
**Hosmer-Lemeshow $\chi^2(8)$**	12.97	16.02	6.64
**Hosmer-Lemeshow p**	.113	.042	.576
**Area under the ROC curve**	0.752	0.759	0.781
**95% CI for the area**	0.703–0.800	0.712–0.807	0.736–0.826
**Tolerance, lowest**	0.912	0.747	0.876
**Variance inflation factor, highest**	1.097	1.339	1.142

***Note**. Analytic n = 403 for every model. Variance inflation factors and tolerances were obtained by re-estimating each predictor set in a linear regression on the same outcome, because collinearity depends on the predictors rather than on the link function; all values are well inside the conventional limits of tolerance above 0.1 and a variance inflation factor below 10. Adding recruitment round (Model 3) improved every index of fit and discrimination. A further model adding round-by-predictor interaction terms to Model 3 produced a significant block ($\chi^2(3) = 12.58$, $p = .006$), driven by the pre-consultation term ($B = 1.339$, $p = .010$); the association ran in the same direction in both rounds but was stronger in round 2*
